# Esophagogastrectomy for Long-Segment Recurrent Esophageal Stricture Following Hyperemesis Gravidarum

**DOI:** 10.7759/cureus.42489

**Published:** 2023-07-26

**Authors:** Lorraine I Chong Tai, Gautam Anand, Satya P Singh

**Affiliations:** 1 Department of Internal Medicine, Broward Health Medical Center, Fort Lauderdale, USA; 2 Department of Gastroenterology, Broward Health Medical Center, Fort Lauderdale, USA

**Keywords:** esophagitis, gastroesophageal reflux disease (gerd), esophogastrectomy, esophagectomy, esophageal stricture, hyperemesis gravidarum

## Abstract

An esophageal stricture is an abnormal tightening of the esophageal lumen. Benign strictures are often caused by gastroesophageal reflux disease (GERD) and are more common in patients over 40 years. When caused by GERD, these strictures develop when acid from the stomach regurgitates into the esophagus, leading to inflammation, fibrosis, and eventual narrowing of the lumen. This case report aims to highlight the importance of obtaining a detailed history in discovering the underlying cause of these strictures. We present a unique case of a young female presenting with dysphagia several months after experiencing Hyperemesis gravidarum. She was found to have a long esophageal stricture that would eventually recur within a few weeks of therapeutic intervention. The pathological report confirmed benign disease, but she ultimately would require surgical intervention for her condition.

## Introduction

Hyperemesis gravidarum is a condition of pregnancy characterized by severe and intractable nausea and vomiting. Complications might include dehydration, weight loss, and ketonuria [1]. Women with a history of hyperemesis gravidarum in a previous pregnancy are more likely to have a recurrence in subsequent pregnancies [2]. Severe cases, such as the one described here, may lead to the development of esophageal strictures. The pathophysiological mechanism for developing stricture is thought to be the same as GERD. Although these strictures are generally benign, severe strictures can cause esophageal obstruction resulting in dysphagia, coughing, regurgitation of solids and liquids, and weight loss. Endoscopic dilation is considered first-line management but for cases in which dilation is unsuccessful, surgical intervention is required [3]. Surgical intervention for benign strictures, as in the case described, is considered uncommon. We present a case of a 27-year-old female who required an esophagogastrectomy for a 10-cm long esophageal stricture. The suspected cause being recurrent reflux of gastric contents after hyperemesis gravidarum.

## Case presentation

Here, we describe a case of a 27-year-old female who presented with complaints of dysphagia for one month. She endorsed dysphagia was initially only to solids but progressively worsened to include both solids and liquids. She was reportedly diagnosed with a cardiac mass at a different hospital about five weeks prior to presentation; however, those medical records were unable to be obtained. Her medical history also included a history of hyperemesis fravidarum in both of her pregnancies as well as type 1 Diabetes. Her most recent pregnancy resulted in an intrauterine fetal demise two months prior to presentation at 20 weeks gestational age. She required hospitalization on six separate occasions for Diabetic Ketoacidosis (DKA) secondary to severe nausea and vomiting from hyperemesis gravidarum. Esophagogram was unable to be completed during prior admission as she was unable to swallow a significant amount of contrast. Cardiac Magnetic Resonance Imaging (MRI) did not show any cardiac mass; however, it was remarkable for retained fluid in the proximal esophagus and esophageal thickening at the gastroesophageal (GE) wall junction.

She underwent an Upper Gastrointestinal (GI) endoscopy which showed severe esophagitis, an upper esophageal ring that was dilated with the scope, a long distal esophageal stricture that was dilated in the mid esophagus, and another very tight stricture in the distal esophagus that was unable to be dilated with a controlled radial expansion (CRE) balloon. She was taken back for a repeat endoscopy the next day where the stricture was successfully dilated with savary dilators. Antral biopsies showed mild chronic gastritis and esophageal biopsies showed ulceration with chronic inflammation and granulation tissue. Helicobacter pylori testing was negative.

**Figure 1:**
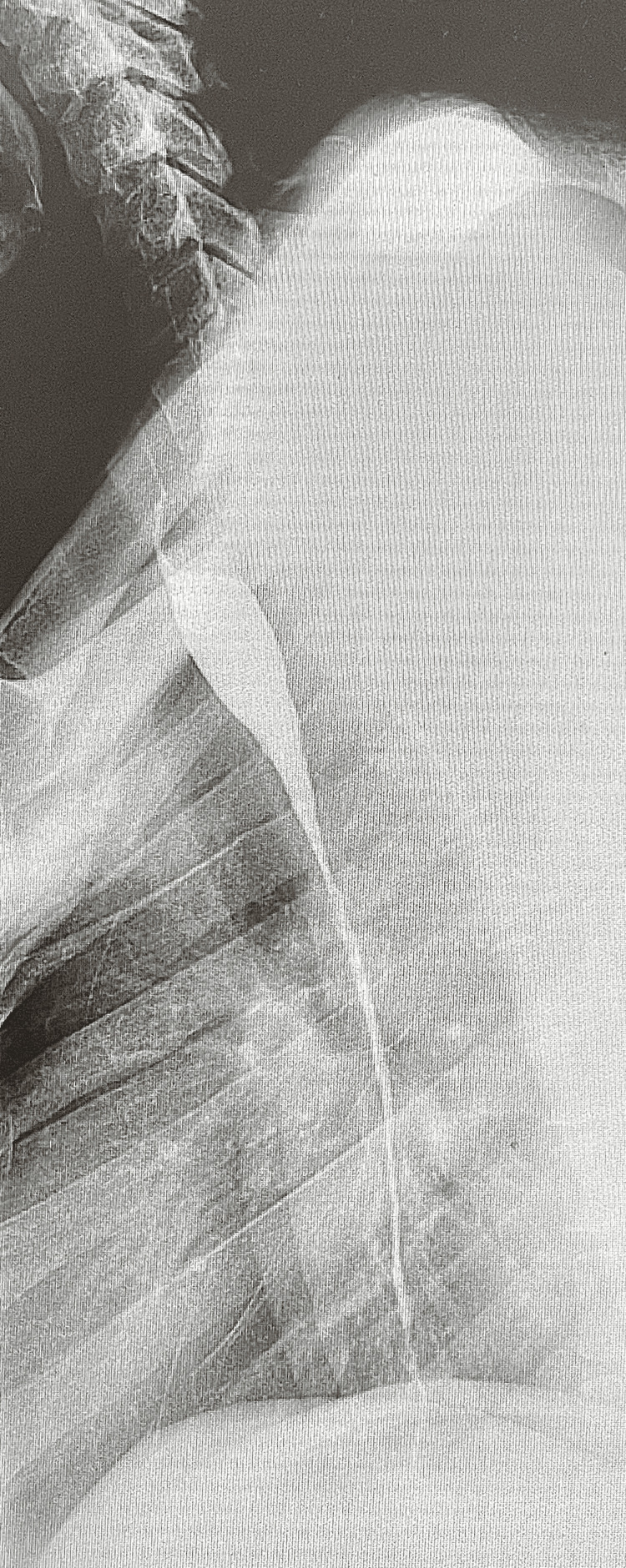
Esophagogram indicating a long segment severe esophageal stricture. No contrast is seen reaching the stomach.

She was subsequently discharged, tolerating a full liquid diet. However, she returned two weeks later with similar complaints of odynophagia and dysphagia to solids and liquids for two days. A Gastrografin swallow study noted a long segment of severe esophageal stricture with no contrast seen reaching the stomach (Figure 1). Endoscopy was repeated and a 10-cm long tight stricture starting around 24 cm of the esophagus was seen. The XP scope could not be passed once again, the scope was removed, and the stricture was once again dilated with savary dilators. There was marked friability of the esophageal mucosa in the distal esophagus. Given her very short response to previous dilation a couple of weeks prior and that the lumen had regressed considerably, she was evaluated by the Thoracic Surgery team for consideration of surgical intervention.

She underwent an open transhiatal esophagogastrectomy, cervical esophagogastrostomy, jejunostomy, and Heineke-Mikulicz pyloroplasty. Intraoperatively, she became hypotensive thought to be secondary to anesthesia. She was transfused with Levophed as well as two units of packed red blood cells (pRBCs) to account for any blood loss during surgery. The patient was intubated postoperatively and transferred to the ICU. She was successfully extubated on post-op day (POD) 1. Supplemental nutrition was provided with total parenteral nutrition (TPN) for five days postoperatively after which she was transitioned to tube feedings through her jejunostomy, which she remained on until her discharge. Intra-operative biopsies taken from the esophagus and stomach showed deep ulceration within the esophagus and the GE junction, chronic inflammation, and fibrosis without any evidence of intestinal metaplasia or dysplasia. Esophagogram confirmed no leak was present on POD 7. She was discharged after a three-week hospital stay. At the time of discharge, she tolerated a full liquid diet while being continued on supplemental tube feeds through jejunostomy. After discharge, she was successfully weaned off of tube feedings and tolerated a regular oral diet.

## Discussion

Esophageal strictures can typically be divided into two categories, either benign or malignant [4]. The majority of benign esophageal strictures result from long-standing GE reflux disease (GERD), accounting for 70% to 80% of adult cases. Other proposed etiologies include radiation or chemotherapy-induced, infectious causes, drug-induced, corrosive substance ingestion, or malignancy [5,6]. In this patient, a review of her biopsy results as well as her social and prior medical history excluded these differentials. Given her lack of history of reflux symptoms prior to pregnancy, as well as the severity of her nausea and vomiting, it appeared most likely her stricture was related to reflux from hyperemesis gravidarum.

Although generally considered a benign condition of pregnancy, serious complications of hyperemesis can develop. These include the development of esophageal strictures leading to obstruction or even esophageal perforation after severe retching [7]. Eroğlu et al. described a case of a 20-year-old female in which hyperemesis gravidarum resulted in esophageal perforation with subcutaneous emphysema for which blunt dissection of the esophagus was then performed [7]. Another case report in 2006 describes a young female who was five months postpartum when she developed dysphagia. She was also found to have a 7-cm long esophageal stricture refractory to endoscopic dilatation with treatment ultimately requiring esophageal resection [8].

It can also be noted that these benign esophageal strictures can develop several months after hyperemesis has resolved. Sato et al. described a case very similar to ours in which the patient developed an esophageal stricture five months postpartum, compared to our patient who developed the stricture two months postpartum. The etiology of hyperemesis gravidarum being the cause of these esophageal strictures could easily be missed. Therefore, these cases emphasize the importance of thorough history taking of prior medical conditions, even if symptoms have already resolved.

Esophageal dilatation is considered first-line treatment for benign esophageal strictures. The major contraindication to dilatation is esophageal perforation [9]. A refractory benign esophageal stricture is defined as the inability to successfully maintain a stricture to a diameter of 14 mm over five sessions at two-week intervals [10]. Esophageal stenting is indicated for refractory esophageal strictures that have failed repeat dilatations [10].

Esophagectomy and reconstruction for benign disease is rare and typically only performed in the following instances: the stricture cannot be dilated, frequent aphagia, intractable refractory esophagitis, those with aspiration pneumonia or in patients whom stricture dilatation is not useful [4]. Esophagectomy for benign diseases typically has good outcomes and can greatly improve the patient’s quality of life [4]. Given the high likelihood of recurrence, this patient opted to undergo esophagectomy rather than undergo esophageal stenting and serial dilatations.

## Conclusions

Hyperemesis gravidarum is considered a rare cause of esophageal strictures with very few cases being documented in medical literature. Making the correlation between hyperemesis gravidarum and the development of these strictures can easily be missed. This case report aims to emphasize the importance of thoroughly reviewing medications, environmental exposures, and prior medical conditions to reveal hyperemesis gravidarum as the etiology. Early recognition and intervention are key. Although invasive, esophagectomy must be considered after esophageal dilation has been proven ineffective.
